# Sleep-wake body temperature regulates tau secretion in mice and correlates with CSF and plasma tau in humans

**DOI:** 10.21203/rs.3.rs-4384494/v1

**Published:** 2024-05-14

**Authors:** Geoffrey Canet, Felipe Da Gama Monteiro, Emma Rocaboy, Sofia Diego-Diaz, Boutheyna Khelaifia, Jessica Kim, Daphne Valencia, Audrey Yin, Hau-Tieng Wu, Jordan Howell, Emily Blank, Francis Laliberté, Nadia Fortin, Emmanuelle Boscher, Parissa Fereydouni-Forouzandeh, Stéphanie Champagne, Isabelle Guisle, Sébastien Hébert, Vincent Pernet, Haiyan Liu, William Lu, Ludovic Debure, David Rapoport, Indu Ayappa, Andrew Varga, Ankit Parekh, Ricardo Osorio, Steve Lacroix, Brendan Lucey, Esther Blessing, Emmanuel Planel

**Affiliations:** Research Center of CHU de Quebec - Laval University; Research Center of CHU de Quebec - Laval University; Research Center of CHU de Quebec - Laval University; Research Center of CHU de Quebec - Laval University; Research Center of CHU de Quebec - Laval University; Department of Psychiatry, NYU Grossman School of Medicine; Mount Sinai Integrative Sleep Center, Division of Pulmonary, Critical Care, and Sleep Medicine, Icahn School of Medicine at Mount Sinai; Department of Psychiatry, NYU Grossman School of Medicine; Department of Psychiatry, NYU Grossman School of Medicine; Department of Psychiatry, NYU Grossman School of Medicine; Department of Psychiatry, NYU Grossman School of Medicine; Research Center of CHU de Quebec - Laval University; Research Center of CHU de Quebec - Laval University; Centre de recherche du CHU de Québec-Université Laval, CHUL, Axe Neurosciences, Faculté de médecine, Département de psychiatrie et de neurosciences, Québec, C; Research Center of CHU de Quebec - Laval University; Research Center of CHU de Quebec - Laval University; Centre de recherche du CHU de Québec – Université Laval, Axe neurosciences, Québec; Department of Neurology, Bern University Hospital; Washington University in St. Louis; Department of Neurology, Washington University School of Medicine; New York University; Mount Sinai Integrative Sleep Center, Division of Pulmonary, Critical Care, and Sleep Medicine, Icahn School of Medicine at Mount Sinai; Mount Sinai Integrative Sleep Center, Division of Pulmonary, Critical Care, and Sleep Medicine, Icahn School of Medicine at Mount Sinai; Mount Sinai Integrative Sleep Center, Division of Pulmonary, Critical Care, and Sleep Medicine, Icahn School of Medicine at Mount Sinai; Mount Sinai Integrative Sleep Center, Division of Pulmonary, Critical Care, and Sleep Medicine, Icahn School of Medicine at Mount Sinai; NYU School of Medicine; Research Center of CHU de Quebec - Laval University; Department of Neurology, Washington University School of Medicine; Department of Psychiatry, NYU Grossman School of Medicine; Centre de recherche du CHU de Québec - Université Laval, Axe neurosciences, Québec

**Keywords:** tau, unconventional protein secretion, sleep-wake cycle, body temperature, Alzheimer’s disease

## Abstract

The sleep-wake cycle regulates interstitial fluid and cerebrospinal fluid (CSF) tau levels in both mouse and human by mechanisms that remain unestablished. Here, we reveal a novel pathway by which wakefulness increases extracellular tau levels in mouse and humans. In mice, higher body temperature (BT) associated with wakefulness and sleep deprivation increased CSF tau. *In vitro*, wakefulness temperatures upregulated tau secretion *via* a temperature-dependent increase in activity and expression of unconventional protein secretion pathway-1 components, namely caspase-3-mediated C-terminal cleavage of tau (TauC3), and membrane expression of PIP_2_ and syndecan-3. In humans, the increase in both CSF and plasma tau levels observed post-wakefulness correlated with BT increase during wakefulness. Our findings suggest sleep-wake variation in BT may contribute to regulating extracellular tau levels, highlighting the importance of thermoregulation in pathways linking sleep disturbance to neurodegeneration, and the potential for thermal intervention to prevent or delay tau-mediated neurodegeneration.

## INTRODUCTION

The intraneuronal accumulation of hyperphosphorylated aggregated tau protein is the pathological hallmark of neurodegenerative tauopathies including Alzheimer’s disease (AD) 1. Both the neuron-to-neuron propagation of tau pathology, and higher levels of cerebrospinal fluid (CSF) and plasma tau correlates with cognitive decline 2. Tau secretion to the extracellular space consequently influences the propagation of tau aggregates in the brain, marking one of the initial steps of pathological tau transmission from diseased to recipient neurons. Further elucidating the key components in pathways underlying tau secretion and key physiological factors regulating their activity may yield insights into therapeutic avenues for slowing the spread of pathological tau.

It is now established that tau, as a leaderless protein, is mainly secreted through the unconventional protein secretion pathway-I (UPS-I), consistent with ~ 90% of extracellular tau being free and unbound to vesicular organelles^3–5^. Key components in this pathway include phosphatidylinositol 4,5-bisphosphate (PIP_2_), which binds to tau at the inner leaflet of the plasma membrane, and heparan sulfate proteoglycans (HSPGs), which facilitate export across cell membrane^4,6^ Furthermore, the main form of extracellular tau is found as its C-terminal-truncated fragment (D421) referred to as TauC3^7–10^. TauC3 cleavage is mediated by caspase-3 and seems to occur intracellularly prior to release^9,11^, and this cleavage at D421 is inhibited by tau phosphorylation at S422^12,13^

Regarding physiological factors regulating tau secretion, previous studies in both mouse and human suggest extracellular tau levels are strongly influenced by the sleep-wake cycle 14, consistent with the established bidirectional link between sleep disturbance and AD neuropathology^15,16^. During wakefulness, CSF and interstitial fluid (ISF) tau levels substantially increase compared to sleep. Sleep deprivation (SD) is associated with elevated tau levels in ISF and CSF 14, while decreasing CSF tau phosphorylation level 10. Moreover, chronic SD reduces tau phosphorylation while promoting its aggregation in AD mice 18. Previous studies suggested that both increased tau secretion and reduced tau clearance contribute to elevated tau levels during waking^10,19^. However, the precise mechanisms by which wakefulness leads to increased ISF and CSF tau remain unknown. We sought to address this question by testing whether tau secretion levels are modulated by the sleep-wake cycle, and investigating mechanisms underlying this modulation. We previously showed sleep-wake differences in tau phosphorylation were driven by fluctuations in core body temperature (BT) during the sleep-wake cycle 20. Furthermore, SD prevented sleep-associated tau phosphorylation by disrupting the normal core BT decrease during sleep 20. Building on these findings, we hypothesized that sleep-wake variations in core BT may drive sleep-wake variation in tau secretion levels by modulating the activity of key components in the UPS-I pathway.

Here, we demonstrate in mice and *in vitro* that variation in CSF and ISF tau levels across the sleep-wake cycle are driven by changes in core BT, owing to temperature-dependent regulatory mechanisms governing tau protein secretion. We found that higher BT, either during wakefulness, SD, or induced by mild-hyperthermia, promotes tau secretion into the CSF *via* the upregulation of UPS-I-related components in mice. We elucidated a specific intracellular pathway involving (i) caspase-3-mediated TauC3 production, (ii) subsequent binding of TauC3 to PIP_2_ at plasma membrane, and (iii) the transmembrane export of TauC3 facilitated by the HSPG family member syndecan-3 (SDC3). In older adults, we found that the rise in BT during wakefulness was positively correlated with the increase in CSF and plasma tau levels.

## RESULTS

### Tau secretion is temperature-dependent

**Figure 1:**
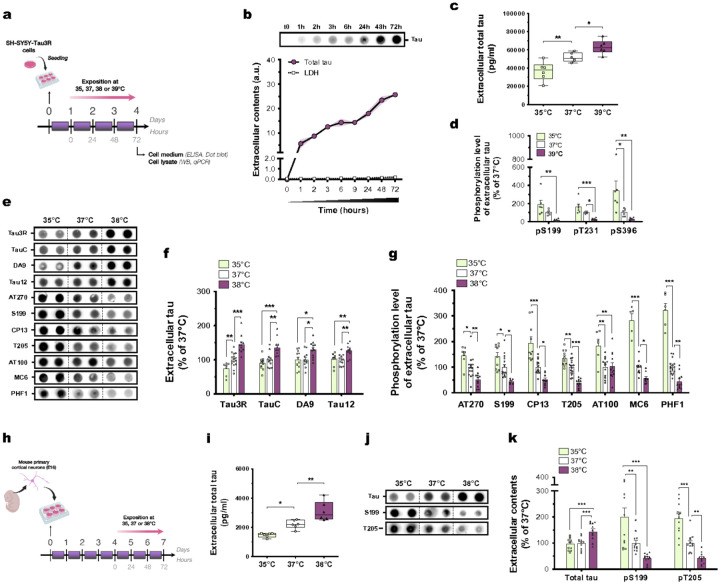
Tau secretion is temperature-dependent in neuronal cells. **(a)** 24 hours after seeding, SH-Tau3R cells were exposed to 35, 37, 38 or 39°C for 72 hours. **(b)** Extracellular accumulation of tau protein (Tau3R; *n* = 6–12; mean ± s.e.m (error envelopes in light purple)) and LDH (n= 4–9; mean ± s.e.m) over-time in cell medium of neurons cultured at 37°C. **(c)** The increase in extracellular tau levels is temperature-dependent in SH-Tau3R cells exposed to 35, 37 or 39°C (*n* = 6; Dunnett’s; box and whiskers with minimum to maximum and median). **(d)** The phosphorylation level of extracellular tau at S199, T231 and S396 is decreased at 39°C compared to 35 or 37°C (*n* = 6; Tukey’s; mean ± s.e.m). **(e, f)** The increase of extracellular tau levels is temperature-dependent (Tau3R, TauC, DA9 and Tau12 antibodies) in SH-Tau3R cells exposed to 35, 37 or 38°C (*n* = 7–16; Tukey’s; mean ± s.e.m). **(e, g)** The phosphorylation level of extracellular tau at AT270, S199, CP13, T205, AT100, MC6 and PHF1 is decreased at 38°C compared to 35 or 37°C (*n* = 7–16; Tukey’s; mean ± s.e.m). **(h)**4 days after seeding, mouse primary cortical neurons were exposed at 35, 37 or 38°C for 72 hours. **(i)** The increase of extracellular tau levels is temperature-dependent in mouse primary neurons exposed to 35, 37 or 38°C (*n*= 6; Dunnett’s; box and whiskers with minimum to maximum and median). **(j, k)** The increase of extracellular tau levels is temperature-dependent (Tau3R antibody), while its phosphorylation level at S199 and T205 is decreased at 38°C compared to 35 or 37°C (*n* = 11–12; Tukey’s; mean ± s.e.m). *p<0.05, **p<0.01 and ***p<0.001.

During wakefulness, CSF and ISF tau levels increase nearly twofold compared to sleep 14. We first investigated whether temperatures simulating BT variations during the sleep-wake cycle could regulate neuronal tau secretion in SH-Tau3R human cells (Fig. 1 a) and in primary mouse cortical cells (Fig. 1 h). We found that extracellular tau levels plateaued after 72 hours at 37°C (Fig. 1 b). Using quantitative ELISA and dot blotting, we then showed that higher temperatures (38°C vs. 35°C) led to a ~ 2-fold increase in tau secretion in both human (Fig. 1c,e,f; Extended Data Fig. 1a) and mouse neuron-like cells (Fig. 1i–k), without inducing cytotoxicity, except for 39°C (Extended Data Fig. 1 b,c). In order to more faithfully replicate physiological temperature variations occurring during a full 24-h sleep-wake cycle, we ensured that shorter exposures (6, 24 and 48 hours) corroborated these findings (Extended Data Fig. 1d). Our prior research indicated that increased core BT induced by sauna-like conditions 21 or wakefulness-like temperature exposure 20, lead to tau dephosphorylation. We replicated these findings, showing a temperature-dependent reduction in phosphorylation levels of both intracellular (Extended Data Fig. 1e,f) and extracellular tau (Fig. 1 d,e,g,j,k) in both SH-Tau3R and mouse primary cells. Overall, exposure to higher temperatures similar to those experienced during wakefulness promotes tau secretion, with the secreted tau species being markedly dephosphorylated.

To assess whether the effect of temperature is specific to tau secretion, we conducted a comparative analysis of extracellular contents for various proteins, including microtubule-associated protein 2 (MAP2), α-synuclein, fibroblast growth factor 2 (FGF2), caspase-1, and Neurofilament light chain (NfL). MAP2 is also a microtubule-associated protein, and NfL is a cytoskeletal protein often used as negative control for extracellular tau^14,22^ Additionally, α-synuclein, FGF2 and caspase-1 are proteins known to be secreted *via* the UPS pathways^23,24^ However, temperature did not affect the secretion profiles of these proteins (Extended Data Fig. 1 g,h), pointing to a specific temperature modulation of tau release though unknown underlying mechanisms.

### Wakefulness temperatures promote tau release through its caspase-3-mediated cleavage

**Figure 2:**
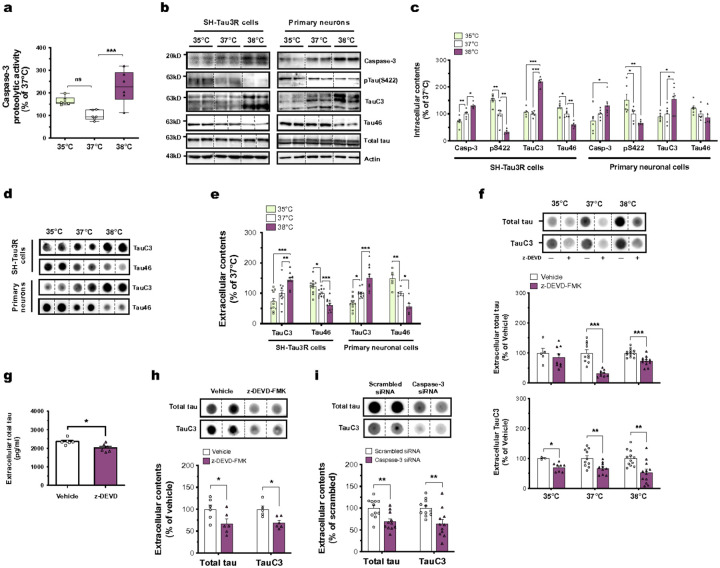
Wakefulness temperatures promote tau release through its caspase-3-mediated cleavage in neuronal cells. **(a)** The increase in the proteolytic activity of caspase-3 is temperature-dependent in SH-Tau3R cells (*n* = 6; Tukey’s; box and whiskers with minimum to maximum and median). **(b, c)** The intracellular expressions of caspase-3, pTau(S422), TauC3 and Tau46 are temperature-dependent in SH-Tau3R (left panels) or in primary neurons (right panels) (*n* = 5–6; Tukey’s; mean ± s.e.m). **(d, e)** The extracellular levels of TauC3 and Tau46 are oppositely temperature-dependent in SH-Tau3R (left panels) or in primary neurons (right panels) (*n* = 6–12; Tukey’s; mean ± s.e.m). **(f)**The inhibition of caspase-3 with z-DEVD-FMK (20 μM) decreases total tau (Tau3R, *n* = 5–12, unpaired t-test) and TauC3 (*n* = 3–8, Mann Whithney) extracellular levels in SH-Tau3R cells exposed to 35, 37 or 38°C (mean ± s.e.m). The inhibition of caspase-3 with z-DEVD-FMK (20 μM) decreases the extracellular levels of **(g)** total tau (ELISA assay, *n* = 6, unpaired t-test), **(h)** Tau3R and TauC3 (Dot blotting, *n* = 5–6; unpaired t-test) (mean ± s.e.m). **(i)** The genetic knockdown of caspase-3 decreases total tau (Tau3R) and TauC3 extracellular levels in SH-Tau3R cells exposed to 37°C (*n* = 11; unpaired t-test; mean ± s.e.m). *p<0.05, **p<0.01 and ***p<0.001. ns: non-significant.

We further sought to identify cellular pathways underlying temperature-dependent effects. Given extracellular tau is predominantly present as the TauC3 proteolytic fragment in AD^7,8^ which is thought to facilitate its secretion 9, we wondered whether temperature affects its caspase-3-mediated cleavage. Our findings revealed that wakefulness temperatures increased caspase-3 activity and protein levels (Fig. 2a–c) in both human and mouse cells, compared to those exposed at 35 or 37°C. Interestingly, this coincided with the intracellular dephosphorylation of tau at S422 (Fig. 2b,c), previously shown to facilitate the caspase-3-mediated cleavage at D421^12,13^. As a result, we observed increased levels of both intra- and extracellular TauC3 in cells exposed to 38°C (Fig. 2b–e). As additional validation, we observed that wakefulness temperatures decreased the levels of intra- and extracellular Tau46 (epitope of 428–441) (Fig. 2b–e), which does not recognize C-term cleaved tau 8. To further substantiate the role of caspase-3 in tau secretion, we observed that its inhibition – either with z-DEVD-FMK pharmacological inhibitor or caspase-3 mRNA-targeting siRNA – significantly decreased extracellular tau release (Fig. 2f–i). Collectively, our data demonstrates that wakefulness temperatures promote caspase-3-mediated cleavage of tau, facilitating its UPS-I mediated secretion.

### Wakefulness temperatures drives TauC3 secretion through SDC3 upregulation

**Figure 3:**
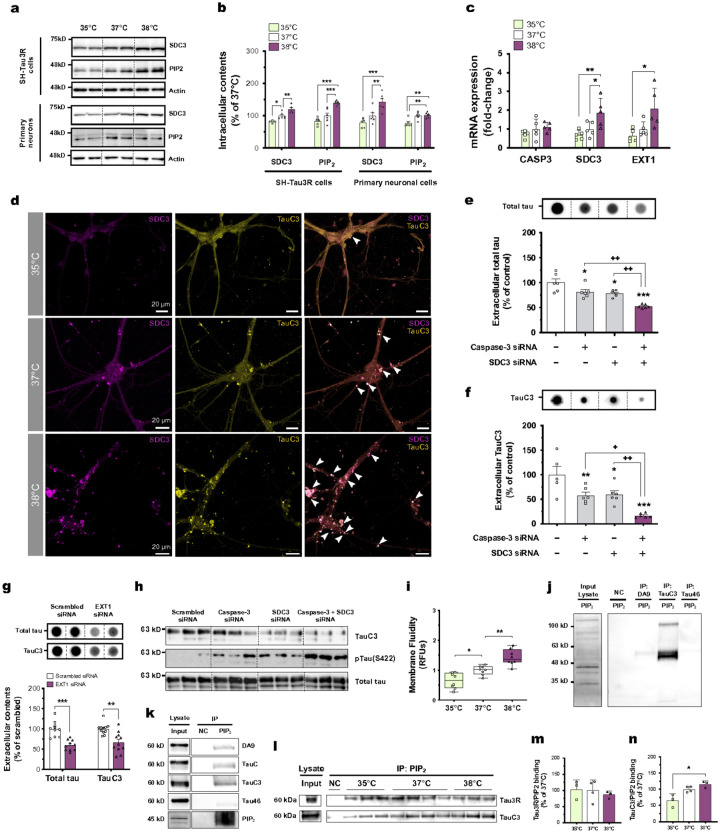
Wakefulness temperatures promote TauC3 interaction with PIP_2_ and SDC3 in neuronal cells **(a, b)** The intracellular expressions of SDC3 and PIP_2_ are temperature-dependent in SH-Tau3R (left panels) or in primary neurons (right panels) (*n* = 6; Tukey’s; mean ± s.e.m). **(c)** The mRNA expression of *SDC3* and *EXT1* genes are temperature-dependent in SH-Tau3R, and *CASP3* mRNA is unchanged (*n* = 5; Tukey’s; mean ± s.e.m). **(d, e)** The genetic knockdown of SDC3 decreases total tau (Tau3R) and TauC3 extracellular levels in SH-Tau3R cells exposed to 37°C, and the co-transfection of caspase-3 siRNA and SDC3 siRNA induces additive effects in the suppression of tau secretion (*n* = 5–6; Tukey’s; mean ± s.e.m). **(f)** The genetic knockdown of EXT1 decreases total tau (Tau3R) and TauC3 extracellular levels in SH-Tau3R cells exposed to 37°C (*n* = 11; unpaired t-test; mean ± s.e.m). **(g)** The genetic knockdown of caspase-3 + SDC3 decreases the intracellular expression of TauC while increasing tau phosphorylation at S422 (representative western blot detection; *n* = 3). **(h)** The increase in membrane fluidity is temperature-dependent in SH-Tau3R cells (*n* = 8; Tukey’s; box and whiskers with minimum to maximum and median). **(i, j)** PIP_2_ displays a better binding affinity for TauC3 rather than full-length tau (DA9 and Tau46 and TauC antibodies) in SH-Tau3R cells exposed to 37°C. NC: negative control, IP: immunoprecipitation. **(k-m)** The increase in binding between PIP2 and TauC3 is temperature-dependent (*n* = 3; Kruskal-Wallis; mean ± s.e.m). *p<0.05, **p<0.01 and ***p<0.001 vs. respective control condition; +p<0.05 and ++p<0.01 vs. indicated condition. **(n)** A merged staining is displayed, showing a temperature-dependent increase of colocalization between SDC3 (purple) and TauC3 (yellow) in primary mouse cortical neurons, and marked with white arrows. Scale bar: 20 μm (magnification of dotted boxes).

HSPGs have been identified as critical components of the UPS-I pathway due to their ability to bind to intracellular proteins and facilitate their direct export across the plasma membrane 25. Ubiquitously expressed on cell surfaces, HSPGs consist of a core proteoglycan with heparan sulfate chains, the elongation of which is facilitated by the glycosyltransferase activity of Exostosin-1 (EXT1) in the brain 26 Among the diverse family of HSPGs, neuronal SDC3 is particularly relevant to AD pathophysiology, promoting the propagation of amyloid pathology and the neuronal uptake of tau^27,28^. To obtain insight into how temperature might drive tau secretion through the UPS-I pathway, we investigated whether temperature influences SDC3 metabolism. We found wakefulness temperatures increased SDC3 and EXT1 protein and mRNA expressions in SH-Tau3R cells and primary neuronal cells (Fig. 3a–c). To further emphasize that TauC3 is highly releasable, we used confocal microscopy and observed a temperature-dependent increase in the merged staining of SDC3 and TauC3, with numerous puncta (SDC3- and TauC3-positive) mainly localized in the soma and in proximal neurites of primary neurons cultured at 38°C (Fig. 3d; Extended Data Fig. 2). As SDC3 might be also involved in tau internalization inside neurons^27,28^, we observed peri-membranous puncta that were TauC3 and SDC3, although in a lower proportion compared to intracellular ones (Fig. 3d; Extended Data Fig. 2).

To confirm the role of SDC3 and EXT1 in tau secretion *via* the UPS-I pathway, we conducted siRNA-mediated knockdown experiments either for SDC3 or EXT1. In both cases, we observed a significant reduction in the extracellular levels of total and cleaved-tau compared to cells transfected with scrambled siRNA (Fig. 3d–f). To further examine the interplay between caspase-3 and SDC3 in tau release, we also simultaneously inhibited the expression of caspase-3 and SDC3 resulting in almost complete suppression of intra- and extracellular TauC3 levels (Fig. 3e,g). Intriguingly, the inhibition of TauC3 expression was associated with a notable increase in tau hyperphosphorylation at S422 (Fig. 3g). These observations collectively emphasize a complementary role of caspase-3 and SDC3 in mediating the extracellular export of TauC3 during wakefulness.

### Wakefulness temperatures facilitate tau recruitment and release at plasma membrane

Due to their lipidic composition, plasma membranes are highly sensitive to temperature fluctuations. An elevation of temperature leads to increased membrane fluidity and permeability in plant and animal cells^29,30^. Based on these findings, we hypothesized that higher temperature exposure might drive tau secretion by optimizing the properties of the plasma membrane to facilitate tau translocation into the extracellular space. We found that SH-Tau3R cells exhibited increased membrane fluidity at wakefulness temperatures (Fig. 3i). Moreover, tau requires sequestration at the inner layer of the plasma membrane through PIP_2_ binding 6. We thus wondered if temperature influences PIP_2_, and showed that its expression was temperature-dependent in both human and mouse neuron-like cells (Fig. 3a,b). Interestingly, the temperature-dependent increase in PIP_2_ expression was previously documented in yeast and plant cells^31,32^ suggesting a highly conserved process.

To explore whether full-length tau and TauC3 have the same affinity for PIP_2_, we performed co-IP using Tau-DA9, TauC3 or Tau46 antibodies and probed for PIP_2_ by Western blot (Fig. 3j), or co-IP using a PIP_2_ antibody to assess the interaction with different tau antibodies (Fig. 3k). In both cases, we observed that PIP_2_ preferentially binds TauC3 rather than full-length tau (Tau46 signal was barely detectable) (Fig. 3j,k). Finally, we demonstrated that an increase in temperature significantly promotes the binding of TauC3 to PIP2, while the binding of full-length tau to PIP2 tends to slightly decrease (Fig. 3l–n). Altogether, these results suggest that wakefulness temperatures promote the UPS-I-mediated secretion of TauC3 by facilitating its interaction with PIP2 and SDC3 at the plasma membrane, thereby triggering its vesicle-free release.

### Wakefulness and sleep deprivation upregulate UPS-I pathway by increasing core BT in mice

**Figure 4:**
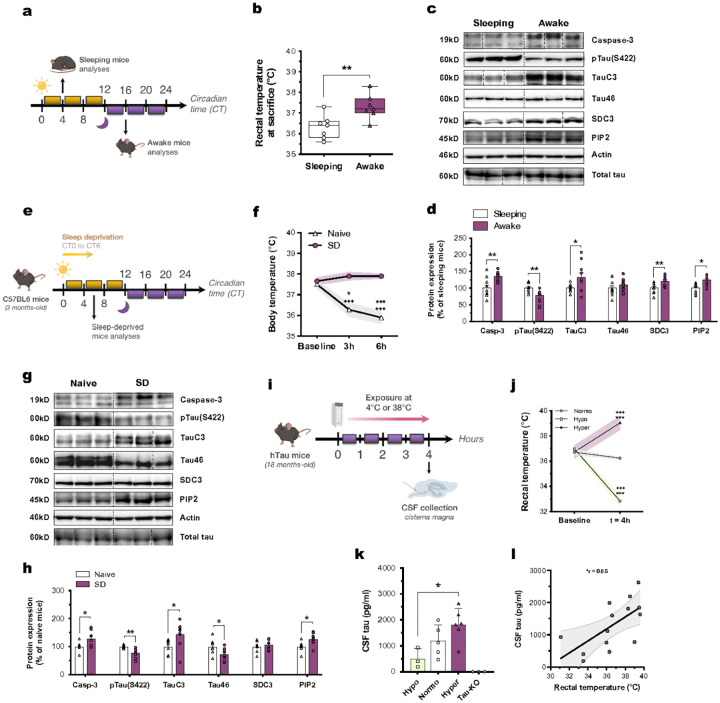
Wakefulness, sleep deprivation and mild-hyperthermia promote the UPS-I-dependent tau release in CSF in mice. **(a)** C57BL6 mice were euthanatized during their sleeping (Circadian time 4, CT4, 10:00 am) or active period (CT16, 8:45 pm, awake group). **(b)** Awake mice display a higher rectal temperature (°C) at sacrifice compared to sleeping mice (*n* = 7; unpaired t-test; box and whiskers with minimum to maximum and median). **(c, d)** The expressions of caspase-3, TauC3, SDC3 and PIP_2_ are increased in the cortices of awake mice compared to sleeping mice, while pTau(S422) is decreased (*n* = 10; unpaired t-test; mean ± s.e.m). **(e)** C57BL6 mice were sleep-deprived (*n*=9) for the first 6 hours of the light period and compared to naive mice (n=7) allowed to sleep without disturbance. **(f)** Sleep-deprivation inhibits the drop in core body temperature (°C) induced by sleep (*n* = 5; Tukey’s; mean ± s.e.m as error envelopes). **(g, h)** The expressions of caspase-3, TauC3, and PIP_2_ are increased in the cortices of sleep-deprived mice compared to naive mice, while pTau(S422) and Tau46 are decreased (unpaired t-test; mean ± s.e.m). **(i, j)** hTau mice were exposed for 4 hours either to 4°C (hypo, *n* = 6) or 38°C (hyper, *n* = 6), and compared to naïve mice (normo, *n* = 5) (Šidák’s; mean ± s.e.m as error envelopes). ***p<0.001 vs. Normo group at baseline; +++p<0.001 vs. respective group at baseline. **(k)** Hyperthermic mice have higher CSF tau levels compared to hypothermic mice (*n* = 3 mice (Normo); *n* = 5 mice (Hypo); *n* = 6 mice (Hyper); Kruskal-Wallis; mean ± s.e.m). **(l)** CSF tau is significantly correlated with rectal body temperature (°C) (Pearson’s correlation; error envelopes in light grey). *p<0.05, **p<0.01 and ***p<0.001.

To assess whether the higher CSF and ISF tau levels during wakefulness and SD 14, are related to natural elevated BT induced by these conditions 20, we analyzed UPS-I-related proteins in the cortex of wild-type mice across sleep vs. wakefulness, or following SD. Awake mice exhibited higher core BT (Fig. 4b), associated with increased cortical expression of caspase-3, TauC3, SDC3 and PIP_2_, along with tau dephosphorylation at S422 (Fig. 4c,d), compared to sleeping mice. Moreover, we observed that the rectal temperature of mice at the time of euthanasia was significantly correlated with the expression levels of caspase-3, pTau(S422), SDC3 and PIP_2_ (Extended Data Fig. 3a-f). We further showed that 6 hours of SD (Fig. 4e) prevented the natural decrease in core BT during sleep (Fig. 4f), and triggered the upregulation of caspase-3, TauC3, and PIP_2_ levels, associated with decreased S422 phosphorylation and Tau46 expression (Fig. 4g,h).

### Mild-hyperthermia increases CSF tau levels in hTau mice

To determine whether induced changes in BT affect CSF tau levels, we subjected hTau mice to hypo- or hyperthermic conditions for 4 hours before CSF collection, and compared to normothermic mice (Fig. 4i,j). We observed that hyperthermic mice exhibited higher CSF tau concentrations (Fig. 4k), and these levels significantly correlated with rectal temperatures recorded after thermal interventions (Fig. 4l). The rise in CSF tau concentrations was associated with increased cortical expression of caspase-3, TauC3, SDC3 and PIP_2_, along with a reduction in tau phosphorylation at S422 and Tau46 expression (Extended Data Fig. 4a,b), all correlating with rectal temperature (Extended Data Fig. 4c-h). These findings collectively suggest that core BT variation influences CSF tau levels through the upregulation of the UPS-I pathway. It emphasizes the pivotal role played by sleep-wake temperature variations in regulating the secretion and the propagation of tau *via* the UPS-I pathway.

### Body temperature correlates with CSF tau but not CSF NfL in humans

**Figure 5:**
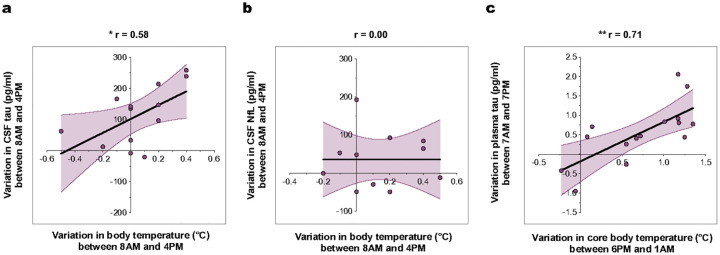
Body temperature correlates with CSF and plasma tau levels in humans. **(a)** The variation in total CSF tau concentrations between 8AM and 4PM is significantly correlated to the variation in oral temperature at the same times (*n* = 13, Pearson’s correlation), **(b)** while no correlation is observed for CSF NfL concentrations and oral temperature (*n* = 11, Pearson’s correlation). **(c)** The variation in total plasma tau concentrations between 7AM and 7PM is significantly correlated to the variation in core body temperature between 6PM and 1AM (*n* = 15, Pearson’s correlation). Standard error bars displayed as error envelopes in light purple, *p<0.05 and **p<0.01. standard error bars displayed as error envelopes in light purple.

To test the relationship between BT and sleep-wake tau dynamics in humans, we utilized two separate data sets from older adults in which BT and tau levels (CSF or plasma) were simultaneously measured at multiple time points across the sleep-wake cycle. We examined the correlation between the magnitude of change in tau levels post-wakefulness (ΔTau) and the concurrent rise in BT during wakefulness (ΔBT). Predefined measurement times were selected to optimize the average ΔBT within the constraints of the available datasets (see Methods). Similar to previous findings in CSF, plasma tau levels were significantly higher in the evening compared to the morning (Extended Data Tables 1 and 2). We found a positive correlation between ΔBT and ΔTau for both CSF tau (r = 0.58, p < 0.05) and plasma tau (r = 0.72, p < 0.005), with no correlation for CSF NfL levels (Fig. 5a–c), and a consistent relationship across CSF and plasma data sets. Specifically, participants exhibiting a large positive ΔTau, i.e., substantially higher afternoon-evening levels compared to morning, also showed a large positive ΔBT. By contrast, participants with negligible or negative ΔTau showed minimal or negative ΔBT (Fig. 5a,c). The observed ΔBT values for the plasma data set agree with our previous study, and represent the first report of correlations between BT and circulating tau in humans. The increase in CSF tau levels with wakefulness vs sleep aligns with previous studies^10,14^ Although diurnal sampling of plasma tau was previously documented in sedentary young adults 33, this is the first report of diurnal dynamics in tau under naturalistic conditions representative of physiological BT variation. Our overall finding of ~15% higher tau in the evening comprised a broad range of ΔTau values that were substantially explained by ΔBT, with similar patterns for CSF. These results supported our *in vivo* and *in vitro* findings, where higher BT during wakefulness drove higher tau secretion.

## DISCUSSION

**Figure 6:**
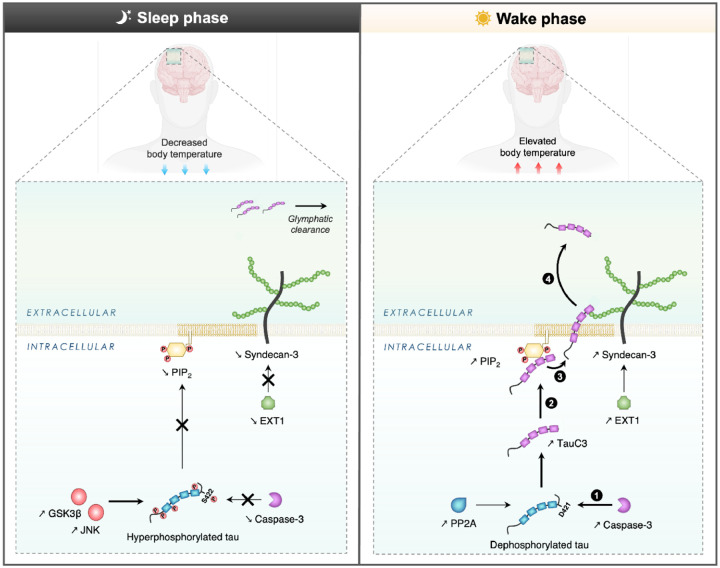
Proposed mechanism to elucidate the regulatory effect of BT on tau secretion *via* the UPS-I pathway during the sleep-wake cycle. During wakefulness, the physiological elevation in BT instigates a series of events triggering tau secretion. **(1)** There is an increase in caspase-3 activity, concomitant with tau dephosphorylation, especially at S422, leading to an augmented cleavage of tau at D421, yielding the TauC3 fragment. **(2)** Subsequently, TauC3 is sequestered at the inner leaflet of the plasma membrane due to its strong affinity binding for PIP_2_. **(3) (4)** The interplay between TauC3 and SDC3 initiates and facilitates the export process across the plasma membrane, that exhibits heightened fluidity and permeability properties during wakefulness. In contrast, during sleep, the decrease in BT inhibits caspase-3 activity and promotes tau hyperphosphorylation at S422, preventing the generation of TauC3. The sleep phase also leads to reduced expression levels of both PIP_2_ and SDC3, as well as to a lower membrane fluidity, resulting in diminished extracellular tau levels.

The present study investigated the influence of BT variation during the sleep-wake cycle upon tau secretion and its underlying regulatory mechanisms (Fig. 6). Our findings indicate that wakefulness temperatures, or conditions affecting core BT such as SD or mild-hyperthermia induction, promotes C-term truncation of tau, leading to its extracellular release through UPS-I pathway. Using *in vitro* and *in vivo* approaches, we identified that the physiological increase in core BT during periods of wakefulness triggers some specific intracellular mechanisms such as (i) the caspase-3-mediated cleavage of tau into TauC3, (ii) the sequestration of TauC3 at plasma membrane *via* its binding to PIP_2_, and (iii) the translocation of TauC3 into the extracellular space facilitated by SDC3, resulting in increased CSF tau levels. This pointed to the involvement of the circadian regulation of BT during the sleep-wake cycle in tau secretion and propagation.

The precise mechanisms underlying tau secretion remain unestablished. Our observation that tau release is modulated in a temperature-dependent manner suggests core BT variation may play a significant role in regulating tau secretion. Holth et al previously showed a twofold rise in ISF and CSF tau levels during wakefulness compared to sleep^14^. Here, we replicated these tau level increases by varying temperature alone within physiological range, with tau doubling at 38°C compared to 35°C.. Excitatory neuronal activity, one of the first identified biological processes capable of increasing tau release^34,35^, is potentiated during wakefulness and depressed during sleep 36. In order to test whether tau release during wakefulness was accounted for by concurrent increases in neuronal activity, Holth *et al*. used tetrodotoxin (TTX) to inhibit neuronal activity, showing that it prevented tau release during SD 14. However, TTX also causes rapid hypothermia^37,38^, pointing to a possible role of temperature in these findings. While it is known that a slight 1°C change in brain temperature is sufficient to alter neuronal excitability and activity^39–41^ we similarly found that a 1°C change alters tau secretion. Given SH cells and mouse primary neurons lack neuronal activity 42, our data strongly suggest that temperature directly regulates UPS-I-mediated tau secretion. However, considering that neuronal activity alone can also drive tau release 34, the interplay between neuronal activity and BT in stimulating tau secretion requires further investigation.

Our study emphasizes tau cleavage into TauC3 as pivotal for secretion, with CSF and extracellular tau mainly present as C-terminally truncated^8,9^. While the administration of a TauC3-specific antibody has been shown to impede tau propagation and seeding 43, the diurnal regulation of these processes remains unknown. We found that wakefulness temperatures induce both tau dephosphorylation at S422, enabling tau cleavage, and upregulation of the caspase-3-mediated TauC3 truncation, leading to its extracellular release. The role of S422 phosphorylation and TauC3 remains debated, with some studies evidencing TauC3 as neuroprotective^44–46^ and others linking TauC3 to neurofibrillary tangle assembly and synaptic toxicity^12,47,48^. Our investigation revealed an inverse relationship between TauC3 and S422 phosphorylation, modulated by physiological sleep-wake fluctuations in core BT. These findings also imply a physiological tau release, consistent with prior studies showing tau secretion does not necessarily result in neuronal pathology spreading^34,35^. In favor to this view, treatment with an anti-pS422 antibody has been shown to reduce AD pathology while increasing plasma tau concentrations in AD mice 49, suggesting that TauC3 might be more prone to brain clearance. However, while wakefulness temperatures induce tau dephosphorylation at multiple epitopes, the relevance of other phosphorylation sites in driving tau secretion remains to be explored.

Here, we have made several novel findings regarding the modulation of tau secretion *via* interactions between multiple temperature-dependent components of the UPS-I pathway. While the UPS-I pathway is known for the release of FGF2 25, α-synuclein 50, and tau^4,5^ our observations of a temperature-dependent effect on tau secretion–without similar changes for others proteins–suggest a unique BT-driven tau secretion pathway. Our results suggest that the temperature-dependent cleavage of tau into TauC3 may serve as an initiating factor for finely modulating its secretion. Notably, the loss of microtubule-binding capacity of TauC3 48 might enhance its availability for the secretion pathway, while wakefulness temperatures facilitate the binding of TauC3 to PIP_2_ at the inner plasma membrane. Prior research has demonstrated that the tau C-terminal domain contains a low-affinity site that affects its interaction with phosphoinositides 51, likely explaining the preferential binding of PIP_2_ to TauC3, given this fragment lacks a portion of the C-terminal domain. Altogether, these findings suggest that higher core BT during wakefulness, SD or mild-hyperthermia, promotes TauC3 binding to PIP_2_ at the plasma membrane, initiating the export process.

The increase in BT during wakefulness appears to also promote the extracellular release of TauC3 by enhancing its interaction with SDC3, facilitating the membrane translocation process. While prior studies reported increased levels of SDC3 in the brain of AD mouse models 28, or following neuronal stimulation 52, this is the first report of its temperature-dependent expression and metabolism. Notably, one study showed that the glycosyltransferase activity of enzymes such as EXT1–required for the elongation of SDC3 sulfate chains–increases with temperature 53. Our study extends these findings, showing that wakefulness temperatures enhance EXT1 mRNA expression, potentially improving SDC3 function. We also observed a substantial intracellular co-localization of SDC3 with TauC3 at wakefulness temperatures, contributing to a better understanding of the mechanisms underlying tau secretion during the sleep-wake cycle.

Our results suggest a pathway by which sleep-wake BT variation may modulate physiological CSF and plasma tau dynamics in human *via* temperature-dependent tau secretion and phosphorylation. However, further research is needed to determine additional temperature-dependent processes. Important candidates include neuronal activity^39–41^, as previously discussed, as well as sleep, known to depend upon body and brain temperature fluctuation 54. Demonstrating a pathway by which temperature influences AD biomarkers *via* sleep, a previous study showed chronic thermoneutral temperature exposure in AD mice reduced amyloid pathology by enhancing slow-wave sleep 55. We also note that bidirectional effects may additionally contribute to the observed relationship between BT and tau dynamics in that early tau pathology in thermoregulatory brain areas may influence BT patterns, as recently shown in mice 56. Finally, in the setting of AD, further research is needed to distinguish between circulating tau derived from unconventional *vs* vesicular secretion 5, or other sources such as impaired degradation and clearance pathways 57, or release after neuronal death 58

Altogether, our findings suggest that sleep-wake BT variation modulates parallel dynamics in tau secretion and phosphorylation, and provide the first evidence associating BT variation with CSF and plasma tau dynamics in human. By extension, our results suggest that impaired thermoregulation as well as BT alteration caused by sleep disturbance may contribute to the pathogenesis of AD and related tauopathies. It is therefore crucial to understand how naturalistic variation in BT over the sleep-wake interval affects CSF and plasma tau levels used for AD diagnosis, particularly in patients with thermoregulatory or sleep deficits. We note that few previous studies in AD patients measured BT variation over the sleep *vs* wake interval–rather most reported BT averaged over the sleep wake cycle, and meta-analysis showed little difference (0.1°C) between AD and controls 59. By contrast, our findings emphasize the importance of assessing BT dynamics over the naturalistic sleep-wake interval in order to understand how BT interacts with tau metabolism. We previously showed that lower waking BT predicted tau pathology, supporting hypotheses that age-associated BT decline may be a risk factor for AD^60–62^. On the other hand, sleep fragmentation or deprivation^16,63^ and increased nocturnal activity^64,65^–both risk factors for, and observed in AD– may prevent the nocturnal BT drop 20, thereby increasing tau secretion and potentially accelerating tau pathogenesis^66,67^

### Conclusions

Our model (Fig. 6) elucidates how core BT regulates tau secretion by driving UPS-I pathway activity during the sleep-wake cycle in healthy individuals. We posit that wakefulness temperatures facilitate physiological tau release, while sleep temperatures inhibit this pathway and increase tau phosphorylation. Further, wakefulness temperatures might facilitate the secretion of dephosphorylated and cleaved tau species which are less toxic, less prone to aggregation and more manageable for clearance and degradation systems within the brain^45,46,49,68^. By contrast during sleep, tau release slows, potentially aiding its clearance via the glymphatic system^68,69^. This model points toward the importance of maintaining and managing the appropriate core BT at the right phase of the sleep-wake cycle, and the potential for age- or AD-related disorder in this pattern to lead to tau pathology. Interestingly, interventions like sauna bathing, which temporarily increase BT 70, are beneficial in reducing AD risk, increasing deep sleep in humans 71, and reducing tau phosphorylation in mice 21. Future studies may examine whether sauna use can delay tau-mediated neurodegeneration by correcting sleep and core BT misalignment associated with thermoregulatory and sleep-disturbances in aging and early AD. Finally, while the physiological role of extracellular tau remains enigmatic, it may act as a signaling molecule, potentially interacting with muscarinic receptors 72. Elucidating the physiological role of tau secretion 73 and understanding the normal function of extracellular tau could inform therapeutic strategies to impede tau pathology propagation.

## METHODS

### Cell culture

In this study, human neuroblastoma cells (SH-SY5Y cells) stably expressing human tau 3 repeat isoform 2+3–10- (designated as SH-Tau3R cells, generously provided by Luc Buée) were used. The SH-Tau3R cells were cultured as previously described 74. Briefly, the cells were grown in DMEM/High glucose medium (11995–065, ThermoFisher), supplemented with 10% bovine growth serum (BGS, heat inactivated, F1051–500ML, Sigma-Aldrich), 1% glutamine (25030081, ThermoFisher), and 1% penicillin/streptomycin (15140–122, ThermoFisher). The cell cultures were maintained in a humidified incubator with 5% CO_2_ at 37°C. The cells were grown either in 10 cm Petri dishes, 6-, 12-, or 96-wells plates.

### Primary culture of neurons

For the primary neuronal culture, cortices of mouse embryos at embryonic day 16 (E15-E17) were used from transgenic mice B6.129S2Emx1tm1(cre)Krj/J, where Emx1-Cre mice were crossed with Red Fluorescent Protein-Lox mice (Jackson Laboratories). Briefly, brains embryos were dissected out, meninges, choroid plexus and hippocampus were removed to avoid contamination and cortices were mechanically and enzymatically disrupted in the presence of trypsin-EDTA 0,25% (Gibco) for 20 min at 37°C. The cell suspension was filtered through a 70 μm cell strainer and plated onto 6-well plates (200,000 cells/well), which were pre-coated with 50 μg/ml poly-D-lysine (A3890401, ThermoFisher), or on coverslips pre-coated with 1 μg/mL polyethylenimine (043896.03, ThermoFisher) and 50 μg/mL poly-D-lysine in 24-well plates (150,000 cells/well). The cells were firstly grown for 2 hours in DMEM/High glucose medium, supplemented with 10% BGS and 1% of streptomycin/penicillin antibiotics in a 5% CO_2_ humidified incubator at 37°C. Then, the culture medium was changed per a growth medium (NeurobasalTM medium (21103–049, ThermoFisher), 1% glutamine (25030081, ThermoFisher), 2% B-27 supplement (17504044, ThermoFisher), 1% N-2 (17502–048, ThermoFisher) and 1% penicillin/streptomycin). The cultures were maintained at 37°C in a humid atmosphere with 5% CO2 and a growth period of 4 days was allowed before any experimental treatment was administered.

### Temperatures exposure and cell treatments

Prior to initiating any treatment, the cell culture medium was replaced with fresh DMEM/High glucose medium (without BSA) or Neurobasal medium, for SH-Tau3R or primary neuronal culture, respectively. Then, the cells were placed in dedicated CO_2_ incubators set to 35, 37, 38 or 39°C for a duration from 6 to 72 hours (Fig.1a, h). To inhibit caspase-3 activity, cells were treated for a period of 72 hours with the selective caspase-3 inhibitor z-DEVD-FMK (A13503; Adooq Biosciences) at a concentration of 20 μM 9 dissolved in a vehicle solution (phosphate-buffered saline (PBS) containing 0.1% of DMSO). Transfection of small interfering RNA was carried out using Lipofectamine^™^ RNAiMAX transfection reagent (13778075, ThermoFisher) according to the manufacturer’s instructions. Briefly, for each transfection, cells were cultured for 72 hours in 1 ml of Opti-MEM (ThermoFisher) containing 40 μl of Lipofectamine^™^, and 100 nmol of respective siRNAs. The following siRNAs were used: Silencer^®^ Pre-designed EXT1 siRNA (ID116802, ThermoFisher), Stealth RNAi^TI^ SDC3 siRNA (HSS145253, ThermoFisher), and SignalSilence^®^ Caspase-3 siRNA (6466S; Cell Signaling). Silencer^™^ select negative control siRNA (4390843, ThermoFisher) was used as the scrambled negative control.

### Animals

In this study, three-months-old C57BL6 (males and females) and 18-month-old hTau (males) mice or their littermate control tau knockout (TKO; males) 75 were used. The hTau mice were generated by crossing mice expressing the 6 isoforms of nonmutated human tau (known as 8c mice) 76 with murine TKO mice 77. The founders of hTau and TKO colonies originated from a C57BL6 background (B6.Cg-Mapttm1(EGFP)Klt-Tg(MAPT)8cPdav/J, Jackson Laboratories). The animals were handled according to procedures endorsed by the “The Animal Care Committee of Universite Laval (CPAUL-3, approbation number: CHU-22–1027)” under the guidelines of the Canadian Council on Animal Care. All mice had access to water and food ad libitum. The mice were housed in a 12 h light/12 h dark cycle, with the lights being turned on at 7:15 am. At the end of each experiment, mice were euthanized through decapitation without anesthesia, as anesthesia leads to tau hyperphosphorylation^78,79^. The brains were promptly removed and cortices were dissected on ice, frozen on liquid nitrogen and stored at −80°C for further analysis

### Sleeping vs. awake mice

Mice were subjected to a continuous period of darkness lasting for 3 days. The determination of subjective day was determined as previously described 20. Briefly, sleeping C57BL6 mice (n=5 males and n=5 females) were euthanized between 10:30 and 11:30 am local time (at Circadian Time 4 (CT4), 16 h after the onset of activity) and active mice (n=5 males and n=5 females) were euthanized between 10:30 and 11:30 pm local time (at CT16, 4 h after the onset of activity) (Fig. 5a). Furthermore, the sleeping criterion corresponded to mice in the nest, in a “resting posture”, as elucidated by Thoman and Carroll: absence of locomotor activity, absence of movement, absence of erect posture 80. The core BT of mice was assessed just before euthanasia with a rectal probe (RET-3, Brain Tree Scientific Inc) connected to a digital thermometer (Thermalert TH5; Physitemp).

### Sleep deprivation

As previously described by our group 20, a subset of C57BL6 mice was intentionally kept awake for the first 6 hours of the light period (sleep deprivation (SD) group, n=9, males and females). Naive mice (n=7, males and females) were allowed to sleep without any disturbance. All mice were euthanatized by decapitation at the end of SD period (Fig. 4e). Prior to SD experiment, a subset of five mice of both groups was abdominally implanted with telemetric probes (BodyCap, Anipill) enabling continuous monitoring of their BT. The baseline BT was assessed the day preceding the SD protocol for the same set of animals.

### Cold and heat exposures

On the day preceding the experiment, hTau mice were individually housed to prevent any mutual heating. For the entire duration of the study, the naive group (n=5) and the negative control TKO (n=3) remained at the standard temperature of the animal facility (22°C). As previously described by our group^21,81^, the two other groups of mice underwent a 4-hour exposure period either at temperature of 4°C (n=3) or 38°C (n=5). The core BT of mice was assessed just prior to euthanasia utilizing a rectal probe (RET-3, Brain Tree Scientific Inc) connected to a digital thermometer (Thermalert TH5; Physitemp).

### CSF collection

The mice were anesthetized with isoflurane and positioned on a stereotaxic instrument. To maintain core BT, a water heating pad was used. Under the observation of a dissection microscope, the subcutaneous tissues and muscles (m. biventer cervicis and m. rectus capitis dorsalis major) were gently separated via blunt dissection utilizing forceps. This separation facilitated the exposure of the dura mater of the cisterna magna. A capillary tube was introduced through the dura mater into the cisterna magna in order to induce the CSF flow into the capillary tube.

### Protein extraction

The samples (cell lysates or mice cortices) were homogenized by sonication in Radioimmunoprecipitation assay (RIPA) buffer, then centrifuged for 20 min at 20,000g at 4°C. The resulting supernatant was collected, and the total protein concentration was assayed (Pierce^™^ BCA Protein Assay Kits, 23225, ThermoFisher). The samples were diluted in sample buffer (NuPAGE LDS; Invitrogen) containing 5% of 2-β-mercapto-ethanol, 1 mM Na_3_VO_4_, 1 mM NaF, 1 mM PMSF, 10 μl/ml of Proteases Inhibitors Cocktail (P8340; Sigma-Aldrich). The samples were then subjected to denaturation for 10 min at 95 °C.

### Western blotting

Western blot analysis was conducted as previously described 82. 10–20 μg of the samples were separated on an SDS-10% polyacrylamide gel and transferred onto nitrocellulose membranes (Amersham Biosciences). The membranes were saturated, hybridized with the appropriate antibodies, and revealed as described in 82. For immunoblots targeting phospho-tau epitopes, the signal was normalized to the total tau protein. Used as a loading control, other proteins were normalized to β-actin. Representative lanes from the immunoblots were exhibited for each specific experimental condition. The dashed lines indicate segments where certain lanes from the same blot were excluded, and the remaining lanes were combined. Brightness levels were adjusted as necessary to enhance visualization and accuracy.

### Antibodies

All antibodies used in this study, in addition to their dilution, are listed in Extended Data Table 3.

### Dot blotting

The cell medium was harvested following appropriate treatments and centrifugated for 10 min at 20,000g at 4°C to remove cell debris. In order to assess extracellular content of proteins by dot blotting, 100 μl of cell medium were deposited onto nitrocellulose membranes (Amersham Biosciences), utilizing a microfiltration blotting apparatus (Bio-Dot Apparatus 1706545, Bio-Rad). The membranes were saturated, hybridized with appropriate antibodies (Extended Data Table 3) and revealed as described in 82. For dot blots targeting phospho-tau epitopes, the signal was normalized to the total tau protein. In the case of other proteins, the normalization was performed relative to the respective extracellular LDH value (CytoTox 96^®^ Non-Radioactive Cytotoxicity Assay, Promega). Representative dots signal was exhibited for each specific experimental condition. The dashed lines indicate segments where certain dots from the same blot were excluded, and the remaining dots were combined. Brightness levels were adjusted as necessary to enhance visualization and accuracy.

### Co-immunoprecipitation

Co-immunoprecipitation (co-IP) analyses were performed to determine interactions between PIP_2_ total tau and TauC3, following manufacturer’s instructions (Pierce^™^ Classic Magnetic IP/Co-IP Kit, 88804, ThermoFisher Scientific). Briefly, SH-Tau3R cells were harvested using lysis buffer, incubated at 4 °C for 5 min, and centrifuged at 13,000g to pellet cellular debris. The supernatants were collected, proteins levels were adjusted to 500 μg and primary antibodies (Extended Data Table 3) were added to samples, except for the negative control (NC) sample. The samples were then incubated overnight at 4°C on a rotating device. Following this step, protein A/G magnetic beads (25 μl) were added to each sample and incubated for 1 hour with agitation at room temperature. The antibody-bound beads were extracted using a magnetic device and washed three times. The beads were dissociated using the elution buffer and separated magnetically. To neutralize the low pH environment, 10 μl of neutralization buffer were added to the supernatant. The resulting sample was diluted with sample buffer (NuPAGE LDS; Invitrogen) containing 5% of 2-β-mercapto-ethanol, 1 mM Na_3_VO_4_, 1 mM NaF, 1 mM PMSF, 10 μl/ml of Proteases Inhibitors Cocktail, and finally boiled at 95°C for 5 minutes. The proteins were analyzed using Western blot analysis.

### Immunocytochemistry

Neurons from primary culture were fixed in PBS 1X (311–010-CL, Multicell) /4% paraformaldehyde (19210 Electron Microscopy Sciences, ThermoFisher)/10% sucrose (S53, ThermoFisher) for 20 min at room temperature. After washing 3 times with PBS 1X, cells were permeabilized in 0.2% Triton X-100 (T8787–100ML, Millipore) in PBS 1X for 30 min at room temperature and blocked with 5% Goat Serum Heat Inactivated (G6767, Millipore) in PBS 1X for 1 hour at room temperature. Then, cells were incubated with the primary antibodies against TauC3 and SDC3 (Extended Data Table 3) in 5% goat serum heat inactivated in PBS 1X at 4°C overnight. After washing 3 times with PBS 1X, the secondaries antibodies (anti-mouse Alexa Fluor 488 diluted at 1:1000 (#A-11029, ThermoFisher) and goat anti-rabbit IgG Alexa Fluor 633 diluted at 1:1000 (#A-21070, ThermoFisher)) were added for 2 hours. After 3 washes with PBS 1X, DAPI (4’,6-diamidino-2-phenylindole, (ThermoFisher) 3,5μL of DAPI in 25 mL PBS 1X) was used for nuclei staining and coverslips were mounted with Fluoromont-G (00-4958-02, Invitrogen). Sections were imaged on a Zeiss LSM800 confocal microscope system equipped with 405, 488, 561 and 640 nm lasers. Confocal images were acquired and mosaics created using the Zen Blue Edition software (v. 2.3, Carl Zeiss).

### Caspase-3 activity assay kit

The SH-Tau3R cells were cultured in 96-well plates and treated according to appropriate experimental conditions (Fig. 1a). The colorimetric caspase-3 Assay Kit (ab39401, abcam) was used to determine the activity of caspase-3, and following manufacturer’s instruction.

### Membrane fluidity

The SH-Tau3R cells were cultured within 96-well plates and subjected to treatment as outlined in the experimental groups (Fig. 1a). The membrane fluidity (ab189819, abcam) was assessed according to manufacturer’s instructions. Briefly, the cells were incubated 1 hour at temperatures of 35°C, 37°C or 38°C in a cell medium supplemented with 5 ^M of Fluorescent Lipid Reagent and 0.08% Pluronic F127. The fluorescence intensity was then measured (Infinite F200, Tecan) at wavelengths of 400nm and 470nm, using the appropriate filter for excitation at 350nm. The recorded fluorescence values were corrected by subtracting the corresponding blanks from each sample, and the fluorescence ratio of excimer emission (470nm) to monomer emission (400nm) was calculated.

### ELISA assays of extracellular tau

Total and phosphorylated tau concentrations within the cell medium were quantified using ELISA kit: Tau (total) Human KHB0041; Tau [pS199] Human KHB7041; Tau [pT231] Human KHB8051; Tau [pS396] Human KHB7031; Tau (total) Mouse KMB7001 (ThermoFisher). Prior to analysis, the samples were suitably diluted in diluent buffer (1:50 for human tau, 1:2 for mouse tau and phospho-tau). The ELISA assays were performed in accordance with the instructions provided by the manufacturer.

### Quantitative PCR

Total RNA was isolated from SH-Tau3R cells using TrizolÒ reagent (Life Technology) in accordance with the manufacturer’s instructions. The quantification of RNA was conducted, and 1 mg of total RNA was used for cDNA synthesis using the iscript^™^ cDNA Synthesis Kit (Biorad), containing an optimal blend of oligo-dT and random primers. For subsequent PCR amplification, 1 μl of the resultant cDNA was used as template. The primer sequence used for the PCR amplification are reported in Extended Data Table 3. The qPCR mix was formulated with 18 μL per 2 μL of 20 ng cDNA. The mix consisted of 0.5 μL of both the forward and reverse primers, 10 μL of SYBR Green PCR Master Mix (Applied Biosystems), and 7.5 μL of nuclease free water. The qPCR program began with a hot start at 95°C for 3 minutes, succeeded by 40 cycles at 95°C for 15 seconds, followed by 60°C for 1 minute, using a LightCycler 480 II apparatus (Roche). The melting curves were evaluated to ensure a single PCR product. To quantify cDNA levels, the comparative 2ΔΔCt method was employed. Ct values corresponding to the target gene were normalized to the Ct values of the house-keeping gene GAPDH (glyceraldehyde 3-phosphate dehydrogenase). The results were expressed as n-fold differences relative to the experimental control.

### Human Studies

#### CSF temperature correlations:

Detailed information about participants, CSF collection, and study design can be found in Lucey et al. 83. Thirteen participants who completed the placebo group of a recently published clinical trial had 6 ml of CSF collected every 2 hours for 36 hours via an indwelling lumbar catheter 83. All participants were cognitively unimpaired and in good general health except for poor sleep efficiency <85% measured by actigraphy. Body temperature was recorded every four hours with a temporal forehead thermometer (Adc Adtemp 427, American diagnostic Corp, United-States). CSF tau forms (T181, S202, T217) were measured by immunoprecipitation/mass spectrometry as previously described 83. NfL protein levels were quantified using the NF-light^™^ ELISA kit (UmanDiagnostics, Umea, Sweden) following the manufacturer’s protocol. The assay’s measurement range is 100 pg/ml to 10,000 pg/ml with a detection threshold of 33 pg/ml. CSF samples were prepared through dilution with an equal volume of Sample Diluent, achieving a 1:100 dilution ratio, to ensure a volume suitable for analysis. The quantitation process entailed the enzymatic conversion of a colorless substrate into a colored product indicative of the NfL concentration in the samples. Absorbance readings were taken at 450 nm with a reference wavelength of 620–650 nm. To ensure consistency, samples with known high levels of NfL (“bloody CSF”) were utilized as positive controls on each plate diluted to 1:1000. CSF tau-181, tau-202, and tau-217 concentrations were averaged at 8AM, 4PM, and 8PM. Differences between temperature, CSF tau levels, and CSF NfL levels were then calculated for use in the analyses, and detailed data for each participant are provided in (Extended Data Table 1). NfL was selected as control protein because its soluble concentration is not affected by sleep-wake activity 14. We selected time points of 8 AM vs 4 PM for post-sleep vs post-wakefulness tau levels, which also corresponded to the minimum and maximum BT, respectively. These intervals were selected based on the following rationale. First, we assumed little delay between tau secretion and appearance of tau in the CSF, meaning that BT taken at the time of CSF collection would roughly reflect brain temperature at the time of tau secretion. Second, we selected 8 am vs 4 pm as the interval that maximized that difference in sleep vs wake temperatures, given that BT was not recorded during sleep, and for the majority of participants, had already begun to drop between 4 pm and 8 pm (Extended Data Table 1).

#### Plasma temperature correlations:

Data were collected from 24 older adults 68.39±5.25 years of age, 17 of whom were female. Subjects were enrolled in cross-sectional study examining the relationship between core BT and plasma and PET AD biomarkers. Subjects were cognitively normal (n=21) or had mild cognitive impairment (n=3) as determined by the clinical dementia rating scale (CDR), and were medically healthy with only mild, or no sleep apnea. Prior to the study, participants were screened with interviews and one week of home actigraphy for sleep-wake disorders including sleep less that 6 hours per night, significant phase advance or phase-delay. Additional exclusion criteria are detailed in 62 and included AD dementia (CDR > 0.5), medical comorbidities and the use of medications that might affect sleep or thermoregulation, major psychiatric disorders and moderate-severe substance use disorders, shift work within the last 6 months, or traveling across 1 or more time zones within 2 weeks of study participation.

The study design was a semi-naturalistic protocol fully detailed in 62. Briefly, participants underwent continuous measurement of core body temperature using an ingestible telemetric device (Cortemp, HQInc) that sampled temperature every 15 seconds with an accuracy of 0.2°C for a minimum of 36 hours spanning 2 nights. During this time, 2 in-lab nocturnal polysomnograms were measured, and participants were free to behave as they chose during the intervening day between the lab nocturnal recordings. The goal of this design was to capture data that most closely represented the typical BT for each participant. Blood draws for plasma tau were collected on four occasions, in the mornings (7:00 am) and evenings (7:00 pm) on both mornings and nights (Extended Data Table 2). Prior to analysis, temperature data were preprocessed to exclude gaps and artifacts as detailed in 62. Data presented for BT-tau correlations comprised tau levels from night 2 and morning 2, given BT was not always measured prior to blood draw on night 1 (Table S3). Paired tau levels and BT data were obtained for 15 subjects (Extended Data Table 2). For BT–tau correlations, the difference between the average BT between 6–7 pm and 1–2 am was calculated. This interval was chosen because these times represented the sample average minimum and maximum BT, and as such their difference maximized the diurnal BT difference, or ΔBT. Plasma sampling times were selected to maximize efficiency in collecting data. Food intake was not regulated, but the morning sample was typically before the morning meal, whereas the evening sample was typically before the evening meal. Concentrations of plasma tau were measured using the neurology 3-PLEX kit and Simoa HD-X instruments (Quanterix, Billerica, MA, USA) at the NYU Alzheimer’s Disease Center Biomarker Core according to the manufacturer’s instructions. Plasma extraction was performed as described previously. Assays were run in duplicate to obtain inter-assay coefficient of variations (CVs). The inter-assay CV was under 20% for all samples.

### Statistical analysis

A minimum of two distinct experiments were carried out for each experimental condition. Prior to conducting each analysis of variance, an assessment of Gaussian distribution was performed, and its validity was confirmed through a Kolmogorov-Smirnov test (utilizing GraphPad Prism 9.0). Depending on the specific analysis, two-tailed t-tests (or Mann-Whitney tests), as well as one- or two-way ANOVAs (or Kruskal-Wallis tests), were applied. Post hoc analyses, involving either Tukey’s or Dunnett’s tests, were subsequently employed. A significance threshold of P < 0.05 was employed to determine statistical significance. The presentation of data incorporated either box and whisker plots (illustrating the range from minimum to maximum values, encompassing the median) or mean ± standard error of the mean (s.e.m). The scatter plots depicted on each graph provide an indication of the number of data points, and detailed statistical information is provided in Extended Data Table 4.

## Data Availability

The raw data supporting the findings of this study are available on: https://drive.google.com/drive/folders/1iQqmgMdC4GJ04ZQDHb0XIj5Jnd32-iZk?usp=driveJink
